# Mining genes involved in the stratification of *Paris Polyphylla* seeds using high-throughput embryo Transcriptome sequencing

**DOI:** 10.1186/1471-2164-14-358

**Published:** 2013-05-29

**Authors:** Jianjun Qi, Na Zheng, Bing Zhang, Peng Sun, Songnian Hu, Wenjuan Xu, Qin Ma, Tingzhou Zhao, Lili Zhou, Mingjian Qin, Xianen Li

**Affiliations:** 1 Institute of Medicinal Plants Development, Peking Union Medical College, Chinese Academy of Medical Sciences, No.151 Malianwa North Road, Beijing, Haidian District 100193, China; 2 School of Traditional Chinese Pharmacy, China Pharmaceutical University, 24 Tongjiaxiang, Nanjing, 210009, China; 3 Beijing Institute of Genomics, Chinese Academy of Sciences, No.7 Beitucheng West Road, Beijing, Chaoyang District, 100029, China; 4 Yunnan BaiYao Group Co. Ltd, 3686 BaiYao Street, Kunming, ChengGong District, 650500, China

**Keywords:** Embryo, Stratification, Seed dormancy, High-throughput sequencing, *Paris polyphylla*

## Abstract

**Background:**

*Paris polyphylla* var. *yunnanensis* is an important medicinal plant. Seed dormancy is one of the main factors restricting artificial cultivation. The molecular mechanisms of seed dormancy remain unclear, and little genomic or transcriptome data are available for this plant.

**Results:**

In this study, massive parallel pyrosequencing on the Roche 454-GS FLX Titanium platform was used to generate a substantial sequence dataset for the *P. polyphylla* embryo*.* 369,496 high quality reads were obtained, ranging from 50 to 1146 bp, with a mean of 219 bp. These reads were assembled into 47,768 unigenes, which included 16,069 contigs and 31,699 singletons. Using BLASTX searches of public databases, 15,757 (32.3%) unique transcripts were identified. Gene Ontology and Cluster of Orthologous Groups of proteins annotations revealed that these transcripts were broadly representative of the *P. polyphylla* embryo transcriptome. The Kyoto Encyclopedia of Genes and Genomes assigned 5961 of the unique sequences to specific metabolic pathways. Relative expression levels analysis showed that eleven phytohormone-related genes and five other genes have different expression patterns in the embryo and endosperm in the seed stratification process.

**Conclusions:**

Gene annotation and quantitative RT-PCR expression analysis identified 464 transcripts that may be involved in phytohormone catabolism and biosynthesis, hormone signal, seed dormancy, seed maturation, cell wall growth and circadian rhythms. In particular, the relative expression analysis of sixteen genes (*CYP707A*, *NCED*, *GA20ox2*, *GA20ox3*, *ABI2*, *PP2C*, *ARP3*, *ARP7*, *IAAH*, *IAAS*, *BRRK*, *DRM*, *ELF1*, *ELF2*, *SFR6*, and *SUS*) in embryo and endosperm and at two temperatures indicated that these related genes may be candidates for clarifying the molecular basis of seed dormancy in *P. polyphlla* var. *yunnanensis*.

## Background

*Paris polyphylla* var. y*unnanensis* (named “Chonglou” in Chinese) is one of the most famous medicinal plants in China. It is a perennial herbaceous plant of the Trilliaceae family and is found in damp, shady woodlands, forests, and bamboo forests. The rhizome of this plant has been developed into traditional Chinese medicines such as “Yunnan BaiYao” and “GongXueNing”, which are used to treat dispersing blood stasis and hemostasis, to activate blood circulation, to alleviate pain, for detoxification, and to reduce swelling, stop bleeding and reduce inflammation [1-3]. *P. polyphylla* var. *yunnanensis* is easily grown in moist, humus-rich soil in woodland conditions, in full or partial shade. However, its cultivation is difficult because of long seeds dormancy and very slow growth from seed. At present, the wild plant is the only source of the rhizome. However, the wild plant has become rare and endangered because of over collection in recent decades. To preserve the natural resources and ensure a stable and renewable source of *P. polyphylla var. yunnanensis* for medical purposes, successful cultivation of seedlings and planting is imperative.

There are two key factors limiting the extended cultivation of *P. polyphylla* var. *yunnanensis*. One is the difficulty of obtaining enough seedlings. Development of the seed embryo stops at the globular stage, about 120 days after fertilization [4]. Thus, seed germination requires a long period of embryo development and release from dormancy. Under natural conditions, the seeds are dormant for 18 months (some are dormant for over 2 years) with about 40% of them germinating [5,6]. Some studies indicated that stratification could speed up the breaking of seed dormancy to about 6 months [7,8]. The other factor is that this plant grows slowly, taking four years from seed to flowering and another three or four years to develop enough for herb harvesting.

Freshly harvested *P*. *polyphylla* var. *yunnanensis* seeds consist of a mesophyll outer layer coat (bright red), an inner hardy coat, a large endosperm and a very small, undeveloped embryo [5]. According to Baskin and Baskin [9,10] and Huang et al. [7], the seeds are of the morphophysiological dormancy (MPD) type and need a long stratification period. To date, research on *P. polyphylla* has focused on seed dormancy release and changes in seed phytohormone content [7,8], seed stratification [4], cultivation methods [11] and phylogeny and classification [12]. There is little research on molecular mechanisms, especially functional gene studies of seed development and dormancy release. In this work, a high-throughput gene mining method using the 454 Genome Sequencer FLX platform was used for embryo transcriptome sequencing of *P. polyphylla* var. *yunnanensis*. Karin et al. [13] suggested that a combination of high-throughput sequencing with more classical methods could greatly advance our knowledge of plant developmental processes. Recently, some studies on plant development using 454 sequencing were successfully conducted [14,15], showing the efficacy of 454 transcriptome sequencing for rapid gene discovery. The aim of this study was to identify transcripts of the embryo that are involved in seed development and dormancy release in stratification process and attempt to explore its molecular mechanism. qRT-PCR analysis was used to compare the expression differences of identified genes between embryo and endosperm to gain more insight into the stratification process. The increased genomic information produced in this study will aid our understanding of the molecular basis of seed development and dormancy release in *P. polyphylla var. yunnanensis.*

## Results

### Sequencing and assembly

**Figure 1:**
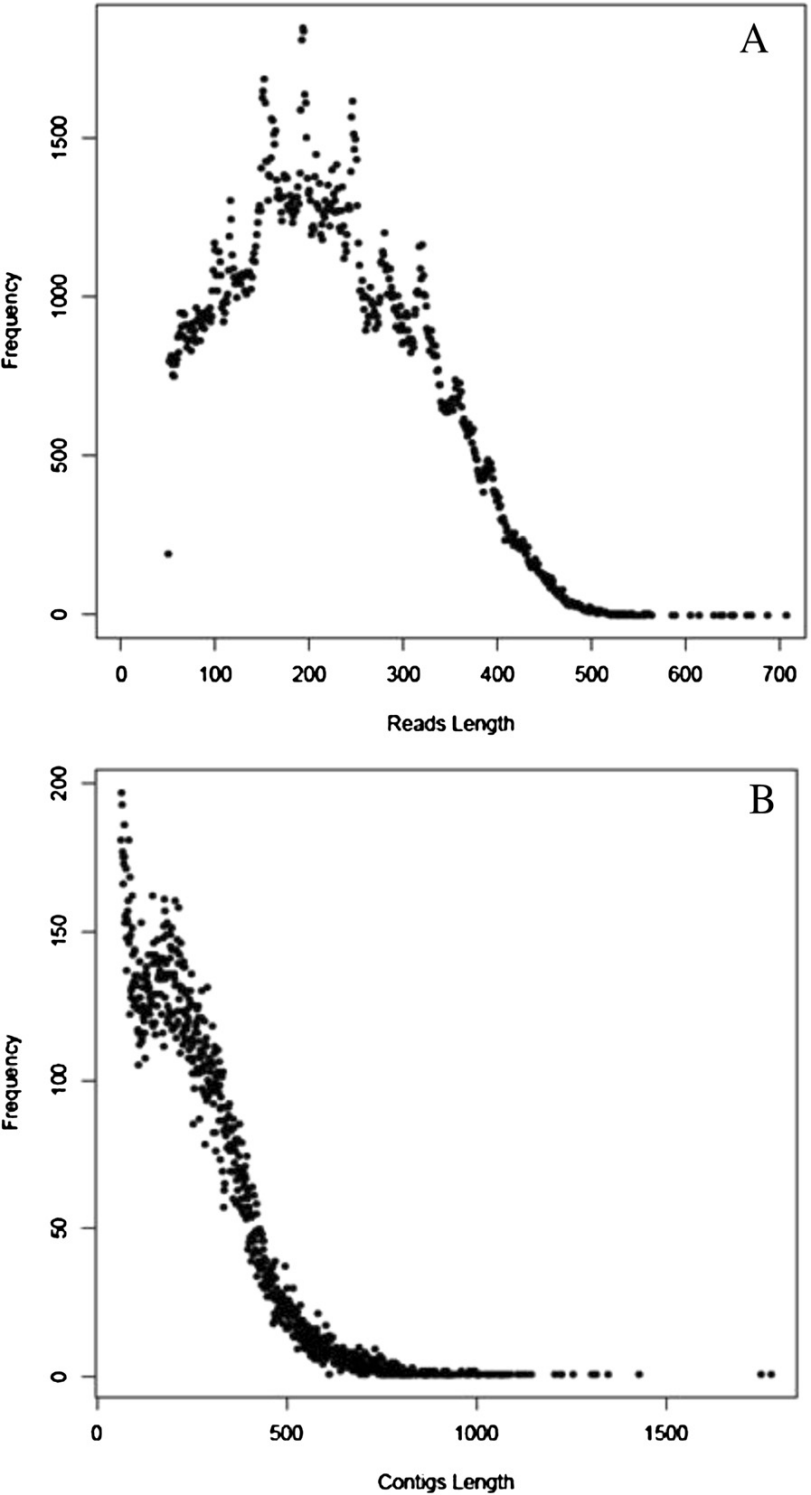
Size distribution of the high quality (HQ) 454 reads and the contigs assembly (A: 454 HQ reads, B: contigs).

We obtained 393,805 reads totaling 88,618,152 bases using the 454 GS FLX Titanium platform. After filtering out the adaptors sequences and removing the short sequences of less than 50 bases, 369,496 (93.8%) high-quality (HQ) reads with lengths ranging from 50 bp to 1146 bp were obtained. The average read length was 219 bp. Using paired-end joining and gap-filling, these reads were assembled into 47,768 unigenes, including 16,069 contigs and 31,699 singletons. The size distribution of these reads and contigs are shown in Figure 1. There were 2,451 (15.3%) contigs with lengths longer than 500 bp, which are considered large contigs. All HQ reads were deposited in the National Center for Biotechnology Information (NCBI) and can be accessed in the Sequence Read Archive (SRA) under the accession number SRX155369. An overview of the sequencing and assembly is given in Table 1.

**Table 1:** Summary of sequencing and assembly

	**Sequence**
Sequencing	
Total bases	80,978,250
Hight-quality (HQ) reads	369,496
Average HQ reads length	219 ±101(bases)
Contigs	
Number of contigs	16069
Contigs larger than 500 bases	2451(15.3%)
Average length of contigs	402 ±160 (bases)
Range of contig lengths	88–1778 (bases)
Singletons	
Number of singletons	31,699
Average length of singletons	237 ±168 (bases)
Range of singleton lengths	50–1146 (bases)
Unigenes	47,768

### Annotation of unigenes

To capture the most informative and complete annotations, all unigenes were first used in BLASTX searches against non-redundant protein database at NCBI. This yielded 15,757 well-identified sequences with E values <10^-5^, which accounted for 32.9% of the total unique sequences. All unique putative transcripts were then subjected to a BLASTX search against the *Arabidopsis* database. Approximately 32.8% of the unique putative transcripts (15,673) were annotated. Subjecting these sequences to a BLASTN search against the NCBI non-redundant nucleotide database yielded 12,663 well-identified sequences with E values <10^-5^, accounting for 26.5% of the total unique sequences. Among the contigs, 30 contained more than 1,000 reads, representing the most abundant transcripts in the 454 cDNA library. The 30 most abundant transcripts included one that encoded early flowering protein. Some transcripts were related with phytochromone and seed ripening, including gibberellic acid stimulated-like (GAST-like), gibberellin oxidase, auxin-repressed protein, ABA 8'-hydroxylase (CYP707A), 9-cis-epoxycarotenoid dioxygenase (NCED), seed maturation protein, late embryogenesis abundant (LEA) protein, xyloglucan endotransglucosylase/hydrolase, dormancy-associated protein (DRM). A number of unique sequences related to seed development and ripening were selected and shown in Table 2. Highly expressed transcripts included cytochrome P450 (60 unigenes), metallothionein-like proteins (105 unigenes), glutathione S-transferase (71 unigenes), and PR10 proteins (22 unigenes).

**Table 2:** Selected genes related to seed embryo development in stratification seeds

**Biological process**		**Gene bank description (gene name)**	**No. of unigene**	**Length (bp)**
Phytohormone related	GA	Gibberellin 2-oxidase (*GA2ox*)	3	124–357
		Gibberellin 20 oxidase (*GA20ox*)	3	147–339
		GA oxidase (*GAox*)	5	232–522
		GAST-like gene product (*GAST-Like*)	7	345–626
		Gibberellin regulated protein; GRAS family transcription factor	8	207–684
		Kaurene oxidase (*KO*)	2	130–213
	ABA	ABA 8′-hydroxylase CYP707A1(*CYP707A1*)	2	256,439
		9-cis-epoxycarotenoid dioxygenase (*NCED*)	2	144,336
		Abscisic stress ripening (*ASR*)	5	176–772
		ABA INSENSITIVE 2 (*ABI2*); Protein phosphatase 2C (*PP2C*)	20	103–669
	Auxin	Auxin-repressed protein (*ARP*)	8	312–806
		SAUR family protein (*SAUR*); Auxin response factor (*ARF*);Auxin influx carrier component (*AUX*)	18	119–727
	IAA	IAA hydrolase (*IAAH*); IAA type protein; Indole-3-acetic acid-amido synthetase (*IAAS*)	13	273–593
	BR	BRASSINOSTEROID INSENSITIVE 1-associated receptor kinase 1 precursor (*BRRK*)	3	121–309
		CPD brassinosteroid C-23 hydroxylase		
	Ethylene	Ethylene-responsive protein (*ERP*)	7	171–391
		Ethylene insensitive (EIN3/EIL)-like transcription regulator 1-aminocyclopropane-1-carboxylic acid oxidase (*ACC*)	3	258–387
Seed maturation related		Late embryogenesis abundant protein (*LEA*)	14	150–709
		Dehydrin (*Dhn*)	13	174–836
		Seed maturation protein (*SMP*)	13	178–1304
		Seed specific protein Bn15D1B (*SCP*)	5	311–646
		Lipid transfer protein precursor (*LTP*)	6	372–727
		Lipid binding protein (*LBP*)	5	126–738
		Somatic embryogenesis receptor-like kinase1 (*SERK*)	6	166–474
		Ripening regulated protein DDTFR19 (*TPS*)	10	221–776
		Trehalose-6-phosphate synthase (*TrPS*)	7	248–494
Cell wall growth related		Xyloglucan endotransglucosylase/hydrolase *(XET*)	19	96–756
		Cell wall invertase (*CIN*)	4	144–654
		Endo-beta mannanase (*MAN*)	5	136–560
		Beta 1,3 glucanase	14	200–647
		Pectin methylesterase (*PME*)	18	109–603
		Expansins (*EXP*)	8	186–639
		Polygalacturonase/inhibitor protein (*PG/PGIP*)	36	184–826
Circadian rhythms		Early flowering protein (*ELF*)	21	256–691
		Sensitive to freezing 6 (*SFR^*)	20	235–542
		Sucrose synthase (*SUS*)	22	122–1313
Flavonol biosynthesis related		Chalcone-flavanone isomerase (*CHI*)	11	98–468
		Chalcone synthase (*CHS*)	17	159–831
Cytochrome P450		Cytochrome P450 (*CYP*)	60	93–525
		Obtusifoliol 14 alpha-demethylase (*CYP51*)	4	152–555
Others		Cell elongation protein (*CEP*)	4	306–721
		Dormancy-associated protein (*DRM*)	3	439–653
		Vaculoar H^+^-translocating inoraganic pyrophosphatase (*VPP*)	10	107–848
Total number of unigenes			464	

### Gene Ontology (GO) and Cluster of Orthologous Groups of Proteins (COG) assignments

The unique putative transcripts were compared with the Universal Protein Resource database (UniPort), formed by uniting the Swiss-Prot, TrEMBL and PIR protein database activities. The BLASTX and the Gene Ontology (GO) assignments of the UniPort proteome produced assignments for 14,601 (30.6%) sequences, matching 9,749 unigenes, and then assigned them to 43 major GO categories for molecular functions, biological processes and cellular components (Additional file 1). The overall distributions suggested that our library sampled widely across sub-categories and provided a good representation of the embryo transcriptome of *P. polyphylla*.

To further evaluate the completeness of our transcriptome library and the effectiveness of our annotation process, we searched the annotated sequences for genes involved in the Cluster of Orthologous Groups of Proteins (COG) classifications. Out of 30,500 nr hits, 5,339 sequences had a COG classification (Additional file 2). Among the 25 COG categories, the cluster for ‘translation, ribosomal structure and biogenesis’ represented the largest group (852, 16.0%), followed by ‘post-translational modification, protein turnover, and chaperones’ (630, 11.8%) and ‘transcription’, which we focused heavily on (207, 3.9%), which were particularly interested in. The categories of extracellular structures (0, 0%), nuclear structures (3, 0.06%) and cell motility (11, 0.2%), represented the smallest groups.

### Functional classification by KEGG

**Figure 2:**
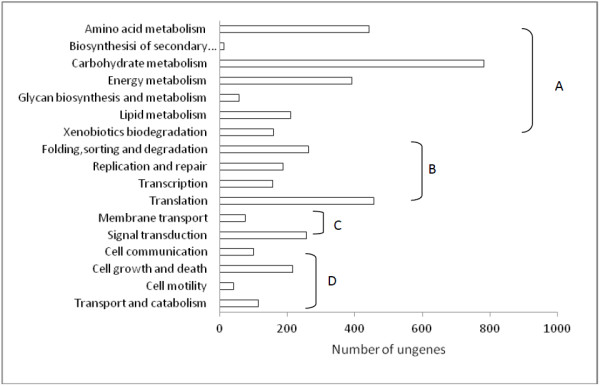
Pathway assignment based on KEGG (A: metabolism; B: genetic information processing; C: environmental information processing; D: cellular processes).

The 47,768 unigenes were compared with the KEGG database using BLASTX with an E-value cutoff of < 10^-5^. Of these unigenes, 15,628 (32.7%) had significant matches in the database. Among those, 5,962 unigenes were assigned to metabolic pathways with an enzyme commission (EC) number; however, 9,666 unigenes were not assigned to any pathways. Figure 2 shows the features of the pathway assignment based on KEGG. Most of the assigned unigenes are involved in the primary metabolites, such as carbohydrate metabolism, amino acid metabolism, energy metabolism and cell growth. Few unigenes participated in secondary metabolites. In particular, 127 unigenes related to phytohormone, sucrose biosynthesis and catabolism were assigned in the KEGG pathway. Some metabolite pathways related with sugar and phytohormone or phytohormone precursor metabolism, including starch and sucrose metabolism, steroid, terpenoid backbone, brassinosteroid and carotenoid biosynthesis are shown in Additional file 3.

### Gene expression analyses of *P. polyphylla* seeds during stratification using qRT-PCR

Using the method of Livak and Schmittgen [16], the level of *GAPDH* mRNA expression was selected as the internal control from four candidate genes (data not shown). The relative expression levels were calculated by comparing the CTs (cycle thresholds) of the target genes with that of the housekeeping *GAPDH* gene, using the 2^-ΔΔCt^ method. Using the Student’s T-test, differences in relative transcript expression levels were compared at *P* < 0.05 level between the embryo and the endosperm at two temperature treatments during seeds stratification. Sixteen primer pairs out of the thirty-six designed primers were successfully amplified a product (the primers shown in Additional file 4).

**Figure 3:**
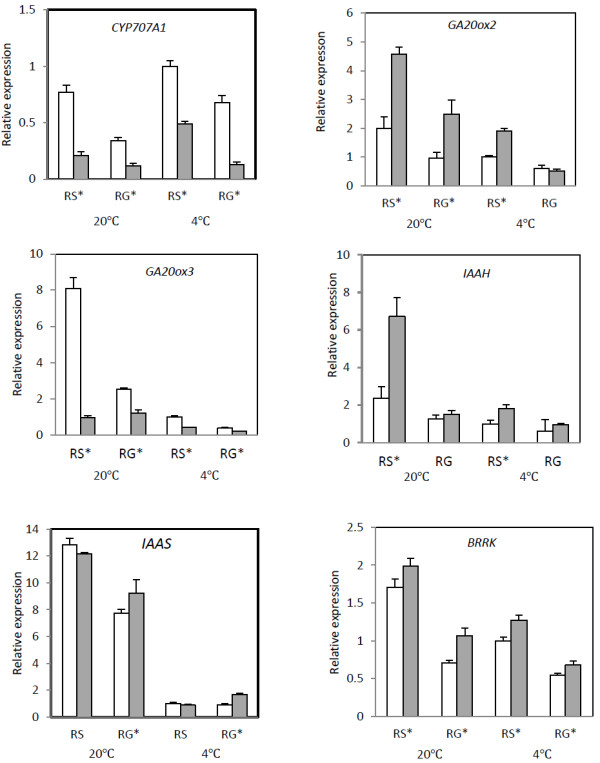
**Relative expression levels analysis of transcripts positively associated with dormancy release (*CYP707A*,*GA20ox2*, *GA20ox3*, *IAAH*, *IAAS*, *BRRK*,) in the Embryo (Em□) and endosperm (En■) under warm (20°C)/cold (4°C) seed stratification processes.** RS, radical just sprouted; RG, radical growing up to 1.5 cm. Expression is shown relative to *GAPDH* as the reference gene. RNA was extracted from three biological samples and qRT-PCR was performed. Data represent means ± SD. * indicate a significant difference between Em and En at *P* < 0.05.

**Figure 4:**
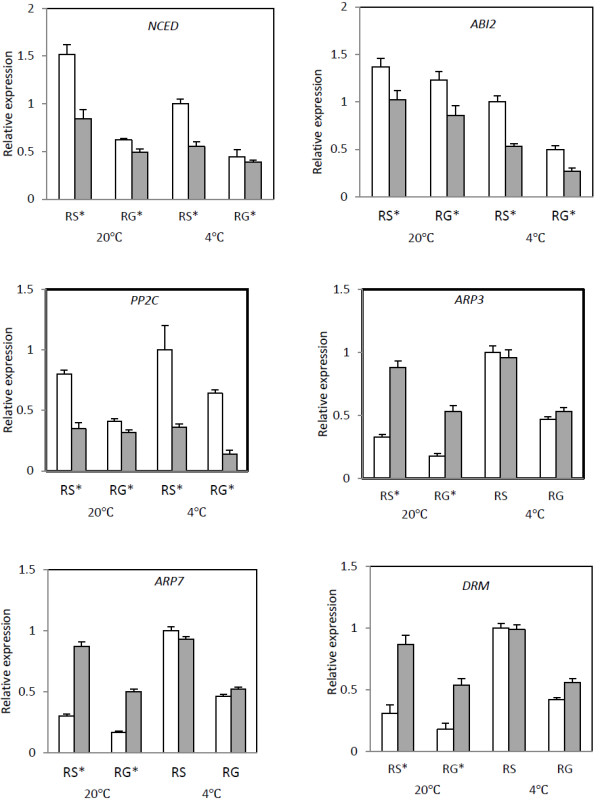
**Relative expression levels analysis of transcripts negatively associated with dormancy release (NCED, ABI2, PP2C, *ARP3*, *ARP7*, *DRM*) levels in the Embryo (Em□) and endosperm (En■) under warm (20°C)/cold (4°C) seed stratification process.** RS, radical just sprouted; RG, radical growing up to 2 cm. Expression is shown relative to *GAPDH* as the reference gene. RNA was extracted from three biological samples and qRT-PCR was performed. Data represent means ± SD. * indicate a significant difference between Em and En at *P* < 0.05.

The hormones abscisic acid (ABA) and gibberellic (GA) are considered central to dormancy and control of germination completion, while auxins are important for plant development and growth. In this work, six ABA and GA related genes (*CYP707A*, *NCED*, *GA20ox2*, *GA20ox3*, *ABI2*, *PP2C*) and five auxins-related genes (*IAAH*, *IAAS*, *ARP3*, *ARP7*, *BRRK*) were studied (Figure 3 and Figure 4). According to the previous studies [17,18], *CYP707A*, *GA20ox2*, *GA20ox3, IAAH*, *IAAS* and *BRRK* might be positively related to dormancy release, in contrast to *NCED*, *ABI2*, *PP2C*, *ARP3* and *ARP7* negatively related with dormancy release.

Figure 3 shows genes that are positively associated with dormancy release. *CYP707A,* which participates in ABA catabolism, showed higher expression levels in the embryo at two stages (radical just sprout (RS) and radical growing up to 1.5 cm (RG)) and at two temperature treatments than within the endosperm (*P* < 0.05). This indicates that ABA catabolism is active at low temperature in both the embryo and endosperm. *GA20ox2* and *GA20ox3*, which are involved in GA biosynthesis, were expressed significantly differently (*P* < 0.05) between the embryo and the endosperm at the two temperature treatments. The mRNA level of the *GA20ox2* in the endosperm was higher than that in the embryo, but the *GA20ox3* expression was higher in the embryo than the endosperm. The expression levels of five auxin related transcripts (IAA hydrolase (IAAH), IAA synthetase (IAAS), auxin-repressed protein (ARP), and brassinosteroid insensitive 1-associated receptor kinase (BRRK)) were successfully determined using qRT-PCR. IAA is a major plant growth hormone that is important for numerous processes throughout plant growth and development. Both IAAH and IAAS participate in increasing plant IAA levels. In this work, the mRNA levels of *IAAH* and *IAAS* were significantly higher (*P* < 0.05) in both the embryo and endosperm under warm stratification compared with cold stratification. In addition, the *IAAH* expression level in endosperms was significantly higher (*P* < 0.05) than in embryos at the two temperatures. The difference in *IAAS* expression level was negligible between the two plant tissues. The *BRRK* gene expression level was positively correlated with temperature, but it expressed higher in endosperms than in embryos (*P* < 0.05).

**Figure 5:**
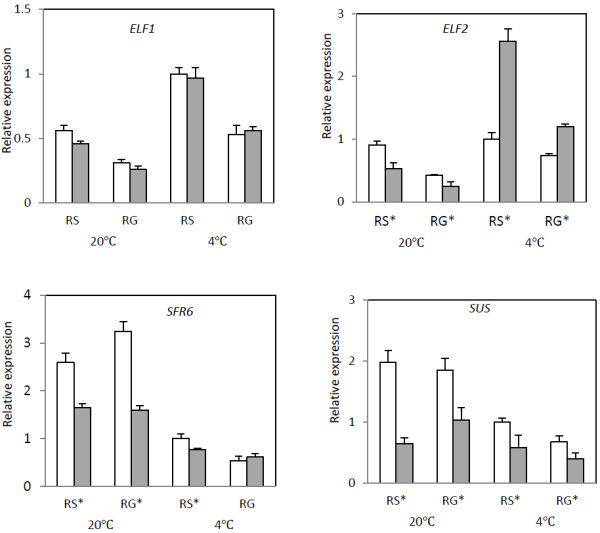
***Paris polyphylla var. yunnannsis *seed (Em, embryo; En, endosperm; Co, cotyledon; Hy, hypocotyls; Ra, radical). A**, the ovule 130d after fertilization; **B**, Freshly harvested seed not at full development (dyed with tetrazole); **C**, **D**, Radicle sprout testa after about 60d stratification.

Figure 4 shows genes that are negatively associated with dormancy release. *NCED*, an ABA biosynthesis key gene, was expressed at an increased level in the embryo relative to the endosperm. It showed decreased expression at the RG stage compared with the RS stage at both temperatures (*P* < 0.05). Among the many protein phosphatease 2C (PP2C) family members in plants, the ABA insensitive 2 (ABI2) belong to the subgroup A and acts as a global negative regulator of ABA signaling. In the presented study, the expression of *ABI2* and a *PP2C* gene were determined by qRT-PCR. The results showed that expression of the two genes had a similar mode, being significantly higher (*P* < 0.05) in the embryo than in the endosperm. *PP2C* showed higher expression in the embryo at 4°C. The relative expression levels of *ARP3* and *ARP7* in the embryo were significantly lower (*P* < 0.05) than in the endosperm at 20°C, but there was no significant difference under cold treatment. These results indicate that the expressions of *ARP3* and *ARP7* were inhibited by warm stratification. In this work, three unigenes were annotated to the same dormancy-associated protein (DRM; AAW02792) and the mRNA level of the *DRM* gene in embryos was significantly inhibited (*P* < 0.05) compared with in the endosperm, under warm stratification, but not under cold stratification. The warm stratification stage is a morphological dormancy release period when the radical, hypocotyls and cotyledon are formed (Figure 5D). Therefore, this result indicates that DRM activity may be highly correlated with morphological dormancy release in *P. polyphylla* seeds.

**Figure 6:**
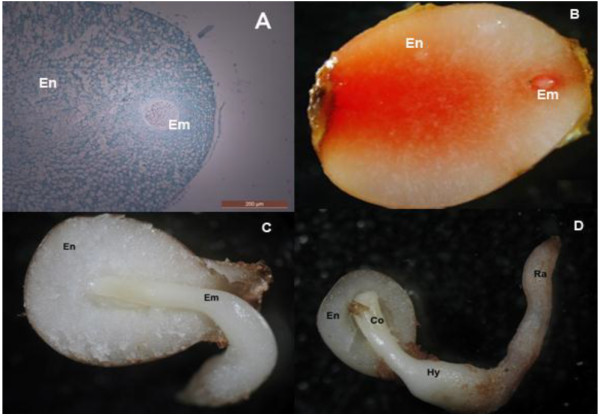
**Relative expression levels analysis of four circadian rhythms related transcipts (*ELF1*, *ELF2*, *SFR6*, *SUS*) in the Embryo (Em□) and endosperm (En■) under warm (20°C)/cold (4°C) seed stratification process. **RS, radical just sprouted; RG, radical growing up to 2 cm. Expression is shown relative to *GAPDH *as the reference gene. RNA was extracted from three biological samples and qRT-PCR was performed. Data represent means ± SD. * indicate a significant difference between Em and En at *P* < 0.05.

Figure 6 shows the qRT-PCR analysis of four plant circadian rhythms related genes, *ELF1 *(*Early flowering protein*), *ELF2*, *SFR6* (*SENSITIVIE TO FREEZING-6*) and *Sus *(*Sucrose*) [19-21]. The relative expression levels of *ELF1* and *ELF2* were significantly higher (*P* < 0.05) under cold stratification than under warm stratification, whether in the embryo and endosperm or at RS and RG stages. There were no differences in the relative expression of *ELF1* in the embryo and endosperm across RNA samples; however, the expression of *ELF2* showed a particular pattern: it was significantly higher expressed (*P* < 0.05) in the endosperm at 4°C stratification but was expressed at a low level in the endosperm at 20°C stratification. The expression levels of *SFR6* and *Sus* were positively correlated with temperature treatments and they both showed higher expressions in embryos than in endosperms (*P* < 0.05). These results indicated that ELF1 and ELF2 might participate in different biological clock regulator processes, while SFR6 and Sucrose might have coincident regulator functions.

## Discussion

### High-throughput transcriptome sequencing is an effectively gene mining method

Freshly matured seeds of *P. polyphylla* var. *yunnanensis* have embryos that are at an undeveloped globular stage. The embryos are very small relative to size of seed, which has a large endosperm (Figure 5A, B). Baskin and Baskin [9] characterized these plant seeds as the Morphophysiological dormancy (MPD) type with a temperature requirement for breaking dormancy and embryo growth. Although cold and/or warm stratification can break dormancy in seeds of many species [9,10], the molecular mechanism of seed dormancy release remains unclear. Nambara and Nonogaki [22] and Mochida and Shinozaki [23] suggested that new methodologies including analysis of transcriptomes, proteomes and metabolomes, might advance our knowledge and understanding of seeds development. Here, we obtained 47,768 unigenes, including 16,069 contigs and 31,699 singletons from the embryo of *Paris polyphylla var. yunnanensis* seeds that had undergone a stratification process, using the Roche 454-GS FLX Titanium platform. Using BLASTX searches of public databases, 15,757 unique transcripts were annotated with specific biological functions. Some transcripts were assigned to important plant physiological and biochemical processes, such as phytohormone biosynthesis and catabolism, seed maturation, cell wall growth, and circadian rhythms regulator, many of which might participate in seed development and dormancy release (Table 2 and Additional file 5). These results demonstrated that *de novo* sequencing and analysis of the *P. polyphylla* seeds during stratification were effective and informative in reflecting the transcript levels in the seed embryo.

However, although we obtained abundant transcripts information, there were two aspects that require further study in future research: (1) Among the annotated 15,757 unigenes, some had functions that were obviously related to seed development and dormancy, others had no specific functions related to any particular biological processes; (2) 32,011 (67%) unigenes were not assigned to any specific functions. To further discover valuable genes in our data base, we consider that combinations of high-throughput sequencing, gene-chip and microarray analysis, and digit gene expression tag profiling with more classical methods (for example gene clone and Southern blotting) could greatly advance our knowledge of plant developmental processes. Recently such combinations were used to study seed-specific transcription factors and gene expression [24,25], and determine the roles of genes in seed dormancy in *Arabidopsis*[26].

### Phytohormone related genes involved in *P. polyphylla* var*. yunnanese* seeds stratification

Compared with orthodox seeds, recalcitrant seeds like *P. polyphylla* undergo little or no maturation dehydration and remain desiccation sensitive during development. However, as in orthodox seeds, Huang et al. [7] and Chen et al. [8] indicated that the ABA levels increased during *P. polyphylla* seed development. According to previous studies [27-29], seed dormancy and germination are controlled primarily by the balance of ABA and GA. ABA is a negative regulator of seed germination, while GA, BR, cytokinins, and ethylene are positively associated with dormancy release. In this work, there were six types of phytohormone-related genes annotated in the database including *GA2ox*, *GA20ox*, *GA3ox*, *CYP707A1*, *NCED*, *BR*, *ACC*, *ACO*, and *IAA*. GA3ox (GA 3-oxidase) and GA20ox (GA 20-oxidase) participated in GA biosynthesis, while GA2ox (GA 2-oxidase) catalyzes the catabolism of biologically active GA and its precursors [29-32]. ABA 8′-hydroxylation (encoded by the *CYP707A* gene family) was shown to play the predominant role in ABA catabolism [33], while NCEDs (9-*cis*-epoxycarotenoid dioxygenase genes) are rate-limiting enzymes in ABA biosynthesis [34,35]. Therefore, *GA3ox, GA20ox* and *CYP707A* are positively associated with dormancy release, while *GA3ox* and *NCEDs* are negatively related. The qRT-PCR analysis indicated that *GA20ox2* and *GA20ox3* expression patterns were very dissimilar in embryos and endosperm even at both temperatures, suggesting they might be different kinds of *GA20ox* genes. These observations are consistent with our annotation results for *GA20ox2* and *GA20ox3* from *Zea mays* (GenBank: ACG35782) and *Glycine max* (GenBank: ACJ76438) (Additional file 5). The *GA2ox* and *GAST-like* (gibberellic acid stimulated-like gene) [36] are also important GA regulator genes; however, qRT-PCR analysis using the designed primers was not successful. In our cDNA library, four unigenes (two contigs for *CYP707A1* and two singletons for *NCED*) associated with ABA metabolism were obtained. Relative expression analysis indicated that *CYP707A* and *NCED* are both expressed both in embryos and endosperm, and are expressed at higher levels in embryos than in endosperm. These result demonstrated that a shift in ABA levels in embryos might be an important factor during seed stratification. However, we only found four transcripts related to ABA metabolism among 47,768 unigenes, which indicated that ABA metabolism is low in the embryos. Footitt et al. [28] suggested that ABA signaling and sensitivity were more likely regulators of dormancy than the absolute level of ABA. We found 22 transcripts of PP2Cs, which are the ABA signaling molecules [37], in our sequence data set. *PP2Cs*, including *ABA-INSENSITIVE1-2* (*ABI1*, *ABI2*), are negative regulators of seed germination [27,37]. The expressions of *PP2C* and *ABI2* genes in this work suggested that ABA signals are more active in embryos than in the endosperm and their expression patterns are consistent with the expressions of *CYP707A* and *NCED*.

IAA hydrolase contributes free IAA to the auxin pool during germination in *Arabidopsis*[38], while IAA synthetase may catalyze the entire pathway of biosynthesis of the major plant growth hormone [39]. The relative genes expression ratios of *IAAH* (IAA hydrolase gene) and *IAAS* (IAA synthetase gene) were compared between embryos and endosperm in *P. polyphylla* at two temperatures. The results indicate that higher *IAAH* and *IAAS* relative expression levels were correlated with stratification temperature and also related to seed morphological dormancy release. We also found that the two expressions of two *ARP* genes were decreased in embryos during warm stratification and were negatively related with *IAAH* and *IAAS* expressions. Park and Han [40] demonstrated that cold treatment abolished the auxin-mediated repression of *RpARP* gene expression and that its expression was negatively associated with hypocotyl elongation. The *Arabidopsis thaliana* SERKs (Somatic embryogenesis receptor kinases) are essential for the early events of BR signaling pathway [41,42]. In the present study, two transcripts of brassinosteroid insensitive 1-associated receptor kinase gene (*BRRK*) and six transcripts of *SERK* gene were found. Relative expression analysis of the *BRRK* indicated differences in transcription levels between embryos and endosperm at two temperatures.

### Other genes associated with *P. polyphylla* var. *yunnanensis* seeds stratification

Dyer [43] identified two dormancy-associated mRNAs with over 3-fold higher expressions in dormant compared with non-dormant embryos of *Avena fatua*. Expression of the dormancy-associated genes *ATS2* and *ATS4* was high in the dry seed and decreased during germination [44]. Using qRT-PCR method, we analyzed a *DRM* gene that is very similar to the dormancy-associated protein gene (GenBank: AAW02792). The expression pattern of *DRM* in this work is similar to that reported by Dyer [43]. Toorop et al. [44] reported high expression in dormant seeds and low expression during germination. However, many similar genes were also found, such as *Oryza sativa* dormancy-associated protein (GenBank: AF467730) and *Zea mays* auxin-repressed 12.5 kDa protein (Eu967389), in nucleotide database. These findings indicated that although only one dormancy related gene (three unigens with same accession no. GenBank: AAW02792) was sequenced in this work, its specific physiological function must be further investigated in the future.

Several genes, such as *CCA1* (*Circadian Clock Associated 1*), *LHY* (*Late Elongated Hypocotyl*), *TOC1* (*Timing of CAB1*), and *GI* (*GIGANTEA*) have been identified as major components of the circadian clock [17,45]. In this work, we obtained two genes, *ELF* (Early Flowering Protein gene) and *SFR6* (Sensitive to Freezing 6 gene) that are related to circadian rhythms. However we did not find *CCA1*, *LHY*, *TOC1*, and *GI*. ELF3 is a novel protein associated with control of plant morphology, flowering time, and circadian rhythms in *Arabidopsis*. Some studies showed that *elf3* mutations cause a light-dependent circadian dysfunction, elongated hypocotyls, and early flowering [20,46]. In this research, 21 unigenes were annotated to *ELF*, which were found in monocots *Elaeis guineensis* (16 unigenes matching GenBank: ACF06553) and *Asparagus officinalis* (five unigenes matching GenBank: AAB09084). Knight et al. [21] indicated that SFR6 is a component of the photoperiodic regulatory pathway. They also observed that clock gene expression and sucrose might act as a regulator of clock function and interacts with SFR6. In the present study, we obtained 20 *SFR6* unigenes, which all annotated to the same *Arabidopsis SFR6* (NP-192401.5) gene, and 22 unigenes that matched sucrose synthase genes. A decline curve of sucrose content variation was found in the *P. polyphylla* seeds stratification process from zero to 100 days and this was accompanied by seed dormancy release (data not shown). In this study, the mRNA expression levels of *ELF1*, *ELF2*, *SFR6* and *SUS* varied in the embryo and endosperm and at both temperatures. These results suggest that ELF, SFR6 and sucrose, as clock components, may participate in the germination of *P. polyphylla* seeds during warm/cold temperature stratification.

LEA proteins might be involved in embryo development or genetic diversity [47,48]. The deduced LEA protein sequences are classified into at least five groups according to their conserved motifs or sequence similarity [49]. Group I to IV LEA proteins are highly hydrophilic, and the remainders are hydrophobic. Dehydrins are group II LEA proteins that contain high glycine levels and are highly hydrophilic [50]. Some dehydrins are constitutively present in vegetative tissues during normal growth [51], but others are induced by tissue water-deficits, such as drought, salinity, low temperature, and seed maturation [52,53]. Recently, in wheat, it was demonstrated that Dehydrin is associated with the maintenance and breaking of seed dormancy, and that ABA affected the expression of *Dehydrin* gene at the transcript level [54]. Sixteen dehydrins transcripts (Additional file 5), which were annotated to seven species of plant dehydrins, were found in this research in our cDNA library.

Cell wall remodeling enzyme, including endo-β-mannanase, β—1, 3- glucanases, expansins, xyloglucan endotransglycosylase, pectin methylesterase, polygalacturonase and cell wall invertase may participate in endosperm weakening and seed germination [55-62]. In the present study, we found 104 unigenes with high similarity to these proteins (Table 2 and Additional file 5). We also found many genes, such as *CHS* (chalcone synthase), *CHI* (chalcone-flavanone isomerase), *F3H* (flavanone 3-hydroxylase), and *DFR* (dihydroflavonol reductase), which were expressed abundantly in the embryo of stratified seeds. Recent research [63] showed that these genes are expressed in the embryo and in the endosperm at germination stages of *Arabidopsis* seeds. However, the physiological role of flavonols in the endosperm is still unclear, though such flavonols might protect the embryo or control seed germination by regulating auxin transport or by acting as scavengers [64].

### The relative expression levels of analyzed genes in seed stratification

In this study, the expression levels of mRNA in different stratification stages or different treatments were altered by less than twofold. We considered that there might be two explanations. (1) Although these genes (GA2ox, GA20ox, CYP707A1, and NCED) are key enzymes involved in a metabolic network, we cannot determine the amount of the final product. Some studies [7,8] showed that ABA content decreased while GA content increased, and the GA/ABA increased by over tenfold when seed stratification was completed. (2) The embryo and the endosperm are two tissues, but they exist in one organ, the seed. Comparisons of gene expression between the embryo and endosperm might show only subtle differences under the same temperature treatment.

## Conclusion

Despite considerable progress in seed biology, the major factors affecting seed dormancy, seed stratification and drying after-ripening remain unclear. Finkelstein et al. [17] and Kucera et al. [18]) have reviewed the role of plant hormones during seed dormancy release and germination, and the molecular aspects of seed dormancy. However, unlike orthodox seeds, the dormancy release of *P. polyphylla* seed is different because of its undeveloped embryo, which requires morphological (cotyledon, hypocotyls and radicle) establishment and physiological dormancy release during the stratification process.

Post-genome methodologies, such as analysis of transcriptomes, proteomes, metabolomes and bioinformatics have advanced our understanding of seed germination. The present study further demonstrates that the high-throughput embryos transcriptome sequencing of *P. polyphylla* seeds is a highly effective method for mining genes that may be involved in seed stratification and dormancy release. A large number of transcriptome data of *P. Polyphylla* seeds have been recently released in the NCBI SRA database under the series identifier SRX155369, which provides an additional resource for the discovery of genes in *P. polyphylla*. Bioinformatics and qRT-PCR analysis help us to identify many important genes, such as *CYP707A*, *NCED*, *GA20ox*, *PP2C*, *SFR6*, *ELF*, *DRM*, which should be studied further to clarify their specific function in seed dormancy release. In addition, there were 67.01% (32,011) unique putative transcripts that were not annotated during BLAST searches against NCBI non-reduntant protein database. This large amount of unidentified sequences should also be studied with a view to annotating new transcripts.

## Methods

### Plant material and RNA preparation

Seeds of *P*. *polyphylla* var. *yunnanensis* were harvested from plants growing in Wuding county, Yunnan Province, China in October 2010. The freshly matured seeds were stored in wet sand in a temperature-controlled incubator at 20°C (warm stratification treatment) for 2 months and then at 4°C (cold stratification treatment) for 2 months. The pulpy outer layer of the seeds coat was removed, and then soaked in water for 24 h prior to the temperature stratification. For the construction of the 454-sequencing cDNA library, three embryo samples at different development stages (radical just sprout testa, radical growing to length 0.5 and 1.5 cm) at two temperature treatments (4 and 20°C) were excised, frozen in liquid nitrogen immediately, and then stored at −80°C until RNA isolation. For qRT-PCR analysis, the embryo and endosperm were sampled at two stages RS (Figure 5C) and RG (Figure 5D), and at the two temperature stratifications.

Total RNA was isolated from 2 mg each of the two temperature treatments using the RNeasy plant kit (BioTeke, Beijing, China). The RNA quality was assessed using a 1% agarose gel and quantified by a GeneQuant100 spectrophotometer (GE Healthcare, Chalfont St Giles, UK) before proceeding. After pollination (13d), the ovules were fixed using FAA (95% alcohol: acetic acid: formalin: water = 10:1:2:7) and made into tablets for microscopic inspection.

### cDNA library construction and pyrosequencing

Approximately, 2 ug of poly(A) RNA was isolated from equal mixtures of the three total RNAs using an Oligotex mRNA Midi Kit (Qiagen, Shanghai China). The long poly(A/T) tails in cDNA may lead to low-quality sequencing reads from the GS FLX system. To overcome this limitation, we designed a modified poly (T) primer with a BsgI site between the adaptor and the poly(T) 5′-AAGCAGTGGTATCAACGCAGAGTACT(20)VN-3′) [65]. For cDNA synthesis, this poly(T) primer was used in combination with the Clontech SMART IV primer. The cDNA was then treated overnight with BsgI (NEB, MA, USA) at a concentration of 5 units/μg of cDNA. This restriction enzyme cut within the poly(A) tail, and greatly increased the quantity and quality of the sequencing reads. For the library, digested cDNA was amplified using PCR Advantage II polymerase (Clontech, Dalian, Chian) and the following thermal profiles were used: 1 min at 95°C; followed by 16 cycles of 95°C for 15 s, 65°C for 30s, and 68°C for 6 min. PCR products (5 ul) were determined by electrophoresis. Approximately 3 ug ds cDNA was sent to the Beijing Institute of Genomics of the Chinese Academy of Sciences (Beijing, China) for pyrosequencing using the 454-GS FLX Titanium Kit.

### Unigene assembly

Using the GS FLX pyrosequencing software, high-quality sequences (> 99.5% accuracy on single base reads) were selected for further processing and assembly. A subsequent filtering step, which included the masking of SMART PCR primer sequences and the removal of sequences shorter than 50 bp, was performed before assembly. The Newbler software 2.3 (provided with the GS FLX sequencer) was used for sequence assembly using the default parameters.

### Sequence annotation

The sequence annotation was based on a set of BLAST searches. The sequences were searched using BLASTX against the NCBI non- redundant protein (nr) database with an E-value cut off of 10^-5^, then the NCBI non- redundant nucleotide (nt) database was searched. The remaining no hit sequences that putatively encoded proteins were searched against the *Arabidopsis* protein database in the *Arabidopsis* Information Resource (TAIR 2.2.8); a typical cutoff value of E < 1.0^-5^ was used. Pathway assignments were carried out according to KEGG mapping. Enzyme commission (EC) numbers were assigned to unique sequences that had BLASTX scores with cutoff values of E < 1.0e5, as determined upon searching the protein databases. The sequences were mapped to the KEGG biochemical pathways according to the EC distribution in the pathway database. We performed a functional classification of the unigenes following the Gene Ontology (GO) scheme. The sequences were annotated by a BLAST search against a series of protein and nucleotide databases, including the curated protein database of Uniprot/SwissProt. The transcripts were classified into 45 GO categories under the major categories of Cellular Component, Molecular Function and Biological Process. A unigene blast was performed using the uniprot database and the annotated GO function. The unigene (congtig and singletons) blast was performed on reference canonical pathways and annotated KEGG pathway.

### Real-time PCR analysis

Total RNA was extracted from embryos and endosperms from warm and cold seed stratification stages using the RNeasy Plant kit (BioTeke, Beijing China). Approximately 1 μg of Dnase I-treated total RNA from each was converted into single-stranded cDNA using a PrimeScript™ RT reagent Kit (Takara, Dalian, China). The cDNA products were then diluted 10-fold with deionized water before use as a template for real-time PCR. Each reaction contained 10 μl 2× SYBR Premix DimerEraser (Takala, Dalian, China), 1 μM each of the forward and reverse primers, and 1 μl of template cDNA. The total reaction volume was 20 μl. PCR amplification was performed under the following conditions: 95°C for 30 s, followed by 40 cycles of 95°C for 5 s and 60°C for 30 s, using CFX96^TM^ Real-Time system (Bio-Rad, USA). Thirty-six unigenes from the 454 data were selected to design qRT-PCR primers and ten housekeeping gene transcripts were selected for design internal control primers (Additional file 4). Each embryo sample in 4°C stratification was selected as a calibrator and the data were presented as the fold change in gene expression normalized to an endogenous reference gene and relative to the calibrator [16]. The qRT-PCR analyses were performed three times with independent RNA samples.

## Abbreviations

ABA: Abscisic acid; BR: Brassinosteroid; COG: Cluster of orthologous groups of proteins; EST: Expression sequence tags; GA: Gibberellins; GO: Gene ontology; IAA: Indo-3 acetic acid; KEGG: Kyoto Encyclopedia of Genes and Genomes.

## Competing interests

The authors declare that they have no competing interests.

## Authors’ contributions

JQ conceived this study, designed and built the cDNA library, participated in data analysis, and drafted the manuscript. NZ participated in seeds stratification, RNA extraction, library construction and data analysis. BZ and SH performed the sequence assembly, alignment and annotations. PS and WX participated in sequence data analysis, the real-time qPCR and the corresponding data analysis. QM and TZ carried out the field management, seed collection and seed stratification. LZ and MQ helped to conceive the study and performed the statistical analysis. XL initiated the project, helped to conceive the study, and participated in the design and coordination. All authors read and approved the final manuscript.

## Supplementary Material

Additional file 1 **Functional annotation of putative unique transcripts from *P. polyphylla *based on GO categories.** Click here for file

Additional file 2 **COG annotations of putative proteins. **All putative proteins were aligned to the COG database and could be classified functionally into at least 25 molecular families. Click here for file

Additional file 3 KEGG pathway involved in phytohormone synthesis such as steroid, terpenoid backbone, brassinosteroid and carotenoid biosynthesis (numbers in red indicate expressed genes). Click here for file

Additional file 4 Primers used in qRT-PCR analysis. Click here for file

Additional file 5 **Possible transcripts involved in seed stratification of *P. polyphylla var. yunnanensis.*** Click here for file
