# Supplementary material for: Mining genes involved in the stratification of Paris Polyphylla seeds using high-throughput embryo Transcriptome sequencing
Source: BMC Genomics. 2013 May 29;14:358. doi: 10.1186/1471-2164-14-358 (PMC3679829; doi:10.1186/1471-2164-14-358)
Supplement: Additional file 1 — Functional annotation of putative unique transcripts from P. polyphylla based on GO categories. [file 1471-2164-14-358-S1.docx]

**Additional file 1** Functional annotation of unique putative transcripts from *P. polyphylla* var. *yunnanensis* based on GO categories.
