# Supplementary material for: Mining genes involved in the stratification of Paris Polyphylla seeds using high-throughput embryo Transcriptome sequencing
Source: BMC Genomics. 2013 May 29;14:358. doi: 10.1186/1471-2164-14-358 (PMC3679829; doi:10.1186/1471-2164-14-358)
Supplement: Additional file 2 — COG annotations of putative proteins. All putative proteins were aligned to the COG database and could be classified functionally into at least 25 molecular families. [file 1471-2164-14-358-S2.docx]

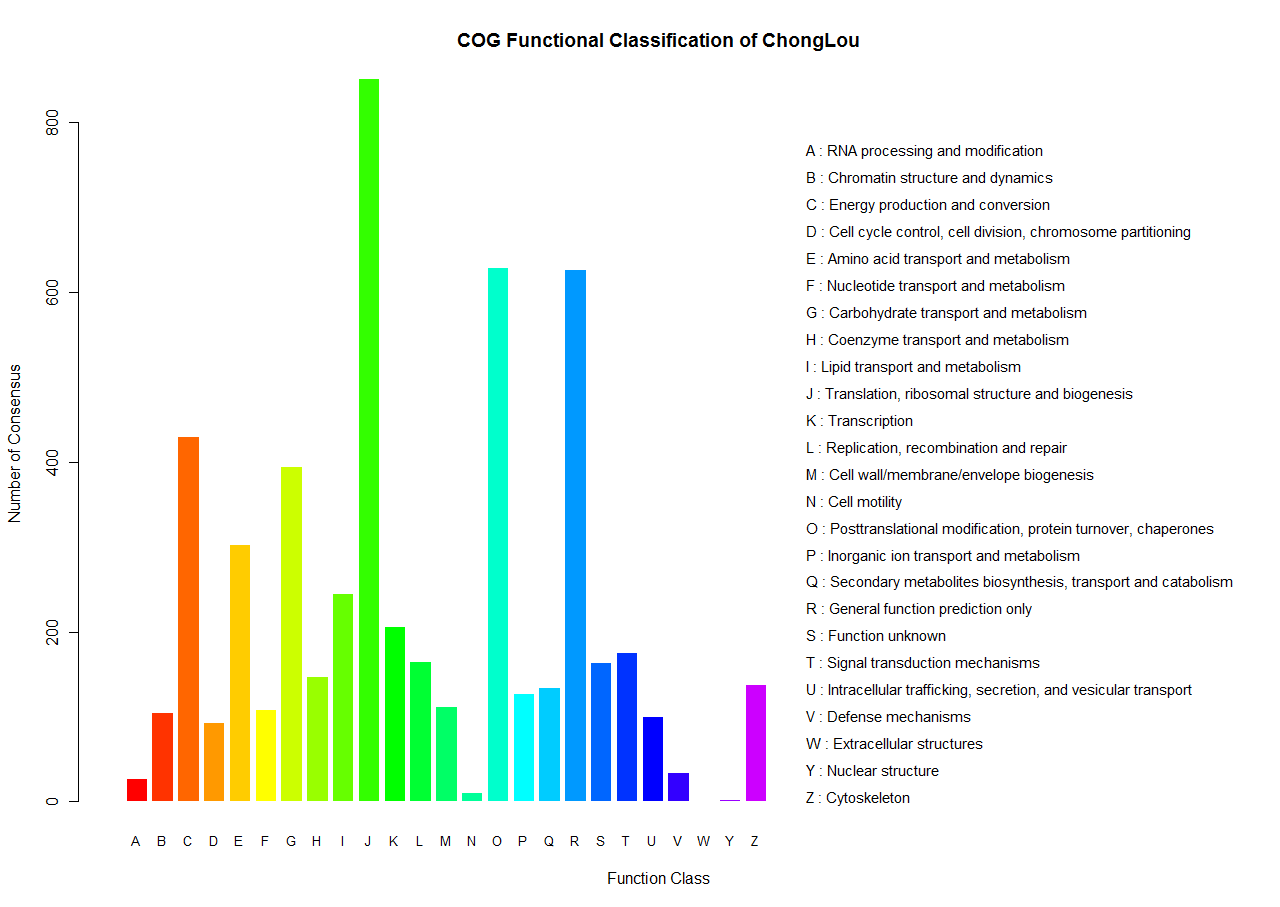


**Additional file 2** COG annotations of putative proteins. All putative proteins were aligned to the COG database and can be classified functionally into at least 25 molecular families.
