# Supplementary material for: Mining genes involved in the stratification of Paris Polyphylla seeds using high-throughput embryo Transcriptome sequencing
Source: BMC Genomics. 2013 May 29;14:358. doi: 10.1186/1471-2164-14-358 (PMC3679829; doi:10.1186/1471-2164-14-358)
Supplement: Additional file 3 — KEGG pathway involved in phytohormone synthesis such as steroid, terpenoid backbone, brassinosteroid and carotenoid biosynthesis (numbers in red indicate expressed genes). [file 1471-2164-14-358-S3.docx]

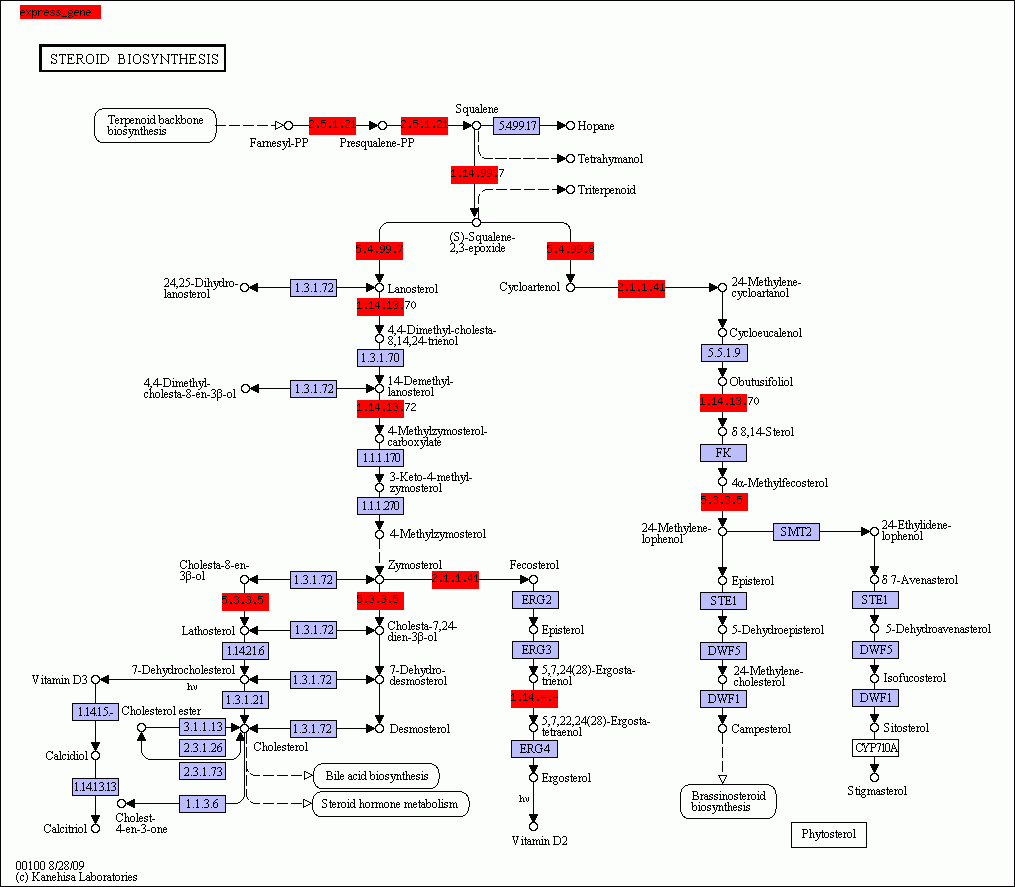


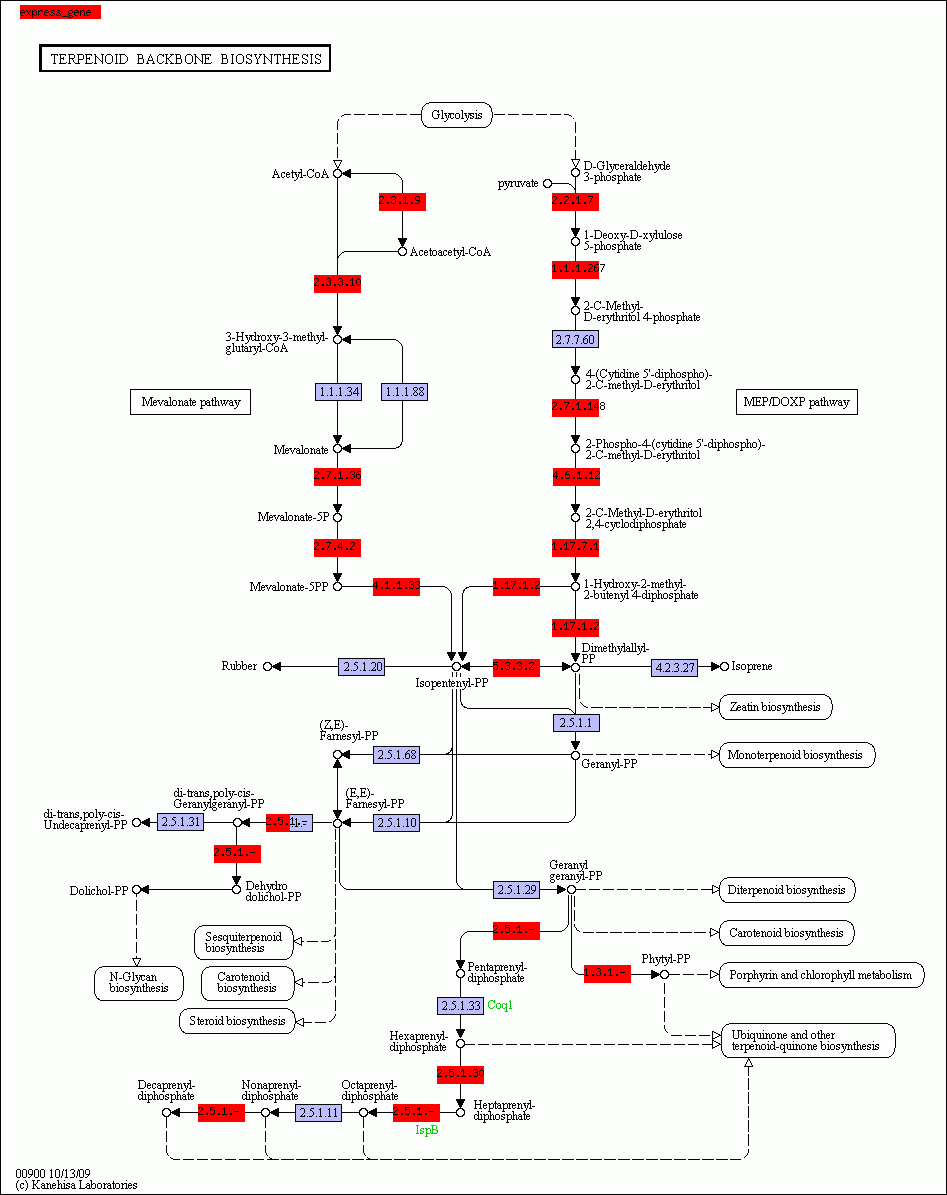


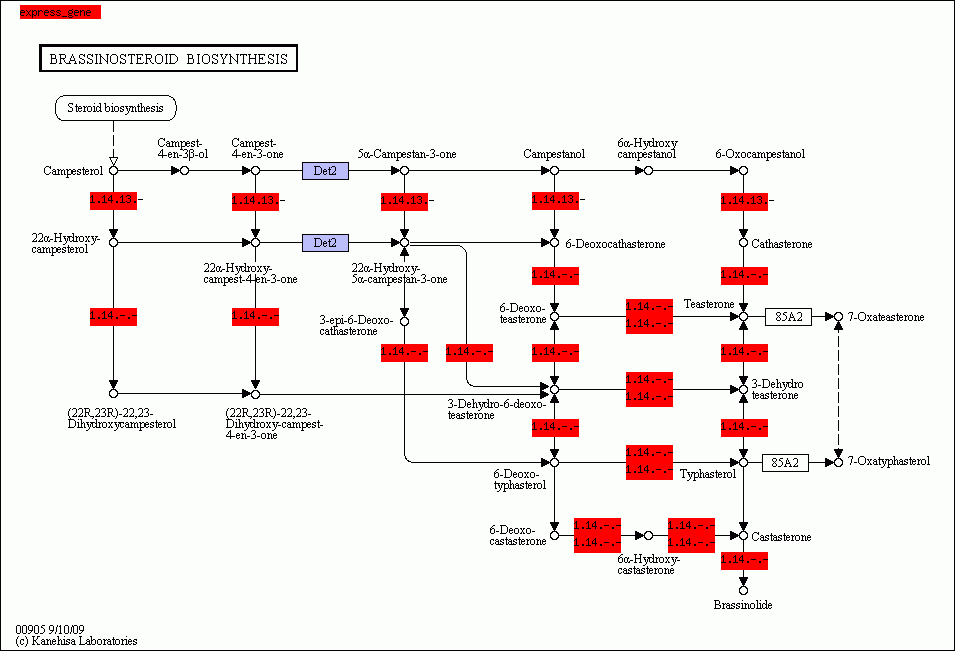


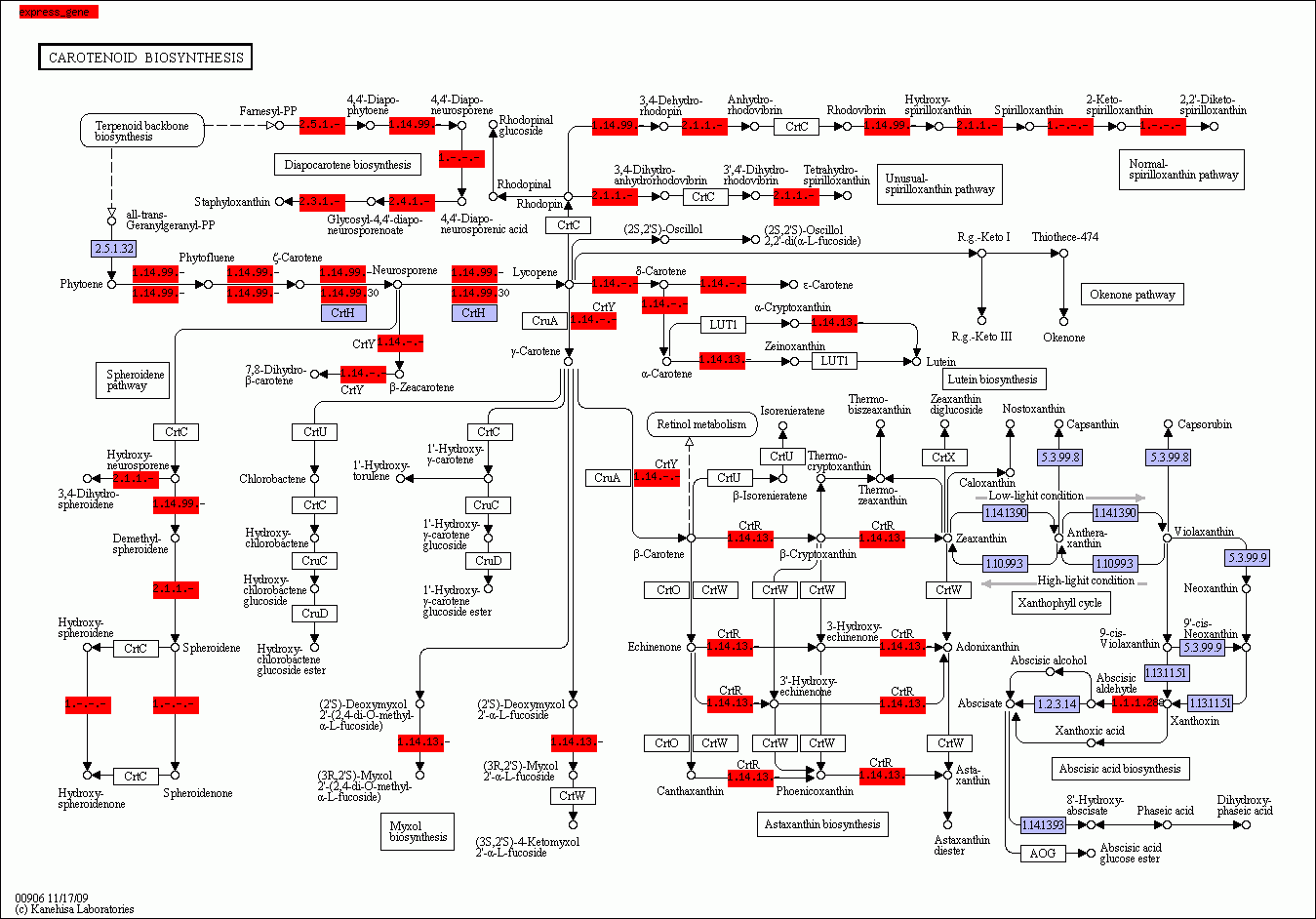


**Additional file 3** KEGG pathway involved in phytohormone synthesis such as steroid, terpenoid backbone, brassinosteroid and carotenoid biosynthesis (numbers in red frame indicated expressed genes ).
